# Abdominal Massage for the Relief of Constipation in People with Parkinson's: A Qualitative Study

**DOI:** 10.1155/2016/4842090

**Published:** 2016-12-08

**Authors:** D. McClurg, K. Walker, P. Aitchison, K. Jamieson, L. Dickinson, L. Paul, S. Hagen, A.-L. Cunnington

**Affiliations:** ^1^Nursing, Midwifery, and Allied Health Professions, Research Unit, Glasgow Caledonian University, Glasgow G4 0BA, UK; ^2^Nursing, Midwifery and Allied Health Professions, Research Unit, Stirling University, Stirling, UK; ^3^School of Medicine, Dentistry & Nursing, Nursing & Health Care School, 59 Oakfield Avenue, Gilmorehill Campus, Glasgow University, Glasgow, UK; ^4^Care of Elderly Department, Glasgow Royal Infirmary, 84 Castle Street, Glasgow G4 0SF, UK

## Abstract

*Objectives.* To explore the experiences of people with Parkinson's (PwP) who suffer from constipation, the impact this has on their lives, and the effect of using lifestyle changes and abdominal massage as a form of constipation management.* Method.* Fourteen semistructured interviews were completed (8 males and 6 females; mean age 72.2 years) at the end of a care programme, which consisted of either lifestyle advice and abdominal massage (intervention group; *n* = 7) or lifestyle advice only (control group; *n* = 7). Data were analysed using constant-comparison techniques and Framework methods. Themes and key quotes were identified to depict major findings.* Findings.* Four key themes were identified: (i) the adverse impact of bowel problems on quality of life; (ii) positive experience of behaviour adjustments: experimentation; (iii) abdominal massage as a dynamic and relaxing tool: experiential learning (intervention group only); (iv) abdominal massage as a contingency plan: hesitation (control group only). Constipation was reported as having a significant impact on quality of life. Participants in both groups perceived lifestyle advice to relieve symptoms. Specific improvements were described in those who also received the abdominal massage.* Conclusions. *Both lifestyle advice and abdominal massage were perceived to be beneficial in relieving symptoms of constipation for PwP.

## 1. Introduction

Constipation is a common nonmotor symptom of neurological conditions [1] including Parkinson's [2, 3]. Constipation is the most common gastrointestinal complaint reported in people with Parkinson's (PwP) and is estimated to impact 27–67% of all sufferers [4, 5]. Furthermore, constipation in Parkinson's has been shown to occur to varying degrees at any time point during disease progression, with epidemiological data indicating that bowel dysfunction can even precede typical Parkinsonian motor symptoms by as much as 20 years [6, 7].

Constipation in Parkinson's is caused by deterioration of the neurological pathways that promote the peristaltic reflex. Reduced peristalsis often exacerbates a slow colonic transit time, resulting in defecatory dysfunction and decreased bowel movement frequency [7]. Lifestyle and individual factors such as poor diet, decreased mobility, general weakness and fatigue, and medication side-effects are also thought to exacerbate bowel dysfunction symptoms [8]. Recommendations for constipation management in Parkinson's therefore include pharmacological treatment, increased physical activity, and dietary modifications such as increased intake of fluids (6–8 glasses water per day), and a high fibre diet [9, 10]. As Parkinson's progresses many patients complain of dysphagia and experience worsening mobility leading to poor diet and difficulty with maintaining levels of activity. Instead, these individuals rely on medicines such as osmotic or stimulant laxatives and stool softeners to help relieve gastrointestinal distress. Though at times effective, laxatives can cause side-effects such as abdominal cramps and diarrhoea, which may lead to faecal incontinence [11].

People with Parkinson's have reported the experience of constipation as distressing, painful, and often debilitating [7]. A UK based National Audit (2015) identified that 80% of patients had been asked about their bladder and bowel symptoms at a routine clinical appointment; however it has also been found that that there is a lack of follow-up and appropriate management with consultations typically focusing on the more visually apparent motor characteristics of the disease [12]. Study of the experience of constipation in PwP is therefore warranted to produce helpful therapeutic approaches, management strategies, and education for PwP who suffer from constipation.

A growing body of research has shown that abdominal massage can reduce the severity of gastrointestinal symptoms, including those who experience chronic constipation [13–15]. Stimulating the parasympathetic division of the autonomic nervous system through a variety of pressured movements is thought to encourage rectal loading by increasing the motility of the muscles and relaxing the sphincters in the gastrointestinal canal. The resulting increase in intra-abdominal pressure promotes peristalsis and bowel sensation [16]. Using abdominal massage as a form of constipation management has also been proposed to reduce laxative use (and thus also their side-effects) [17, 18], improve health related quality of life (QoL) [19, 20], and ease the substantial cost of constipation-related-medicines to primary care [21].

Abdominal massage has been shown to be a safe, effective, and noninvasive form of bowel management in the general population [14, 22, 23], as well as people with multiple sclerosis and stroke [5, 13, 24–26]. It is therefore plausible that PwP who suffer from constipation may also benefit from using abdominal massage as a form of constipation management and this was explored in our feasibility studies [27, 28].

However the experience of living with constipation is inadequately described in the literature and particularly within neurological populations. In one of the few qualitative studies identified, McClurg and colleagues explored the impact of constipation on the QoL of people with multiple sclerosis (MS) [29]. Using phenomenological methodology, the authors highlighted that constipation had a significant impact on the QoL of some people with MS, with themes of decreased self-esteem, loss of control, and reluctance to talk about bowel problems, which was often linked to social isolation.

This study was a cohort study of a prospective two-group (intervention = abdominal massage and advice; control = advice only) single blind randomised controlled feasibility study that aimed to explore the effects of lifestyle advice and abdominal massage on constipation in PwP [27]. The study period was 10 weeks, with base-line outcome assessment (Week 0), 6 weeks of intervention with assessment (Week 6), and final outcome assessment 4 weeks later (Week 10).

*Intervention Group.* The intervention group were asked to self-administer or have a carer administer a 10-minute abdominal massage. The abdominal massage was demonstrated to the patient and/or their carer in their own home and a research nurse visited the patient weekly to offer support on the massage and on the suggested lifestyle changes. Step-by-step written instructions for the abdominal massage were provided with an accompanying DVD. Lifestyle advice, incorporated in a leaflet, included increasing awareness of the importance of fluid intake, fruit and vegetable consumption, physical activity levels, and varying one's position on the toilet.

*Control Group*. Those in the control group were also visited weekly for the 6-week intervention period by the research nurse to offer support around the suggested lifestyle changes as described above. This group was also offered a brief abdominal massage training session and given the DVD at the 10-week follow-up visit following completion of the final outcome measures [27].

It was concluded from the quantitative analysis that abdominal massage as an adjunct to treatment of constipation offers a potentially beneficial intervention to PwP.

This is the first study to explore the views and experiences of PwP in terms of abdominal massage and constipation and aims to explore the experiences of PwP and constipation, as well as the impact that this has on their lives and the effect of using lifestyle advice and abdominal massage as a form of constipation management. As a feasibility study this information is important in going on to design a fully powered randomised controlled study and implementation should be proved effective.

## 2. Methods

### 2.1. Study Design and Sample

An exploratory, qualitative research design was adopted to align with the aims of this study.

### 2.2. Ethical Approval

The study received ethical approval from the West of Scotland Research Ethics 10/S1001/11 and management approval from NHS Greater Glasgow & Clyde R&D GN10GE070. All participants received both oral and written information about the qualitative strand of the study during the initial consultation for the feasibility trial [27]. Informed consent was obtained for participants who wished to take part and the voluntary nature of the study was continually declared. Confidentiality and anonymity were assured. Raw data were stored in a locked filing cabinet and password-protected computer and the study investigators had sole access to data.

### 2.3. Data Collection

Participants in both the intervention and control groups were invited for interview at the end of the pilot study to gain an appreciation of their experiences of being constipated, how it impacted on their QoL and their views on taking part in the study. A number of participants included a family member in their medication management (some of whom also applied the massage to the participant if necessary), and in these scenarios both the patients and their carer/family member were present at interview. A research assistant (PA) who had not been involved in the intervention delivery undertook the interviews by telephone. The topics explored were description of Parkinson's symptoms and their impact on life, impact of constipation on life, management of symptoms, experience of taking part in the study and their perceptions of the effect of the lifestyle advice, and/or abdominal massage on their constipation (see Appendix A.). Interviews ranged from 11 to 31 minutes, were digitally recorded, and were then transcribed verbatim. PA checked transcripts for accuracy before coding and analysis.

### 2.4. Data Analysis

Throughout the data collection process, data were analysed using the constant-comparative technique [30]. DM and PA reviewed and compared interview transcripts regularly, which enabled the identification of emergent themes for exploration in subsequent interviews. Further data management and analysis was approached using the “Framework” method [31]. Familiarisation with data enabled construction of a first level coding framework and was informed by (1)* a priori* research questions underpinning the qualitative element of the study, (2) topics and issues introduced by researchers during the interviews, and (3) recurring themes emerging from interviews with participants. PA conducted this process for each transcript. Initial “indexing” was reviewed by KW, who identified a number of additional emergent codes or themes reflecting patients' experiences of constipation, abdominal massage and bowel management advice. The KW and PA contributed to descriptive analysis, interpretation of indexed data and manuscript preparation with the aid of thematic charts to compare themes within and across the intervention and control groups. In order to ensure validity of interpretation, a sample of indexed data was selected and reviewed by the first author (DM). Key themes and quotes were identified to depict major findings.

## 3. Results

Thirty-two PWP took part in the study from which 14 completed semistructured interviews, 2–4 weeks after completing Week-10 outcome assessments (intervention group *n* = 7; control group *n* = 7). The sample interviewed included 8 males and 6 females with a mean age of 72.2 years. Interviews were conducted either face-to-face (8 interviews) or by telephone (4 interviews). The interview sample was purposively selected to provide a broad range of demographics and equal numbers from the intervention and control group.

This study aimed to explore the experience of constipation in PwP and the feasibility and impact of lifestyle advice and abdominal massage as an intervention within this population. Four main themes emerged from the analysis:* (i) the adverse impact of bowel problems on participants' quality of life; (ii) positive experience of behaviour adjustments: experimentation; (iii) abdominal massage as a dynamic and relaxing tool: experiential learning (intervention group only); (iv) abdominal massage as a contingency plan: hesitation (control group only)* (see Appendix B). The themes discussed are narrated by direct quotations from participants, with an exemplar given for each theme. Numerical values have been assigned to each participant to protect their identities.

### 3.1. The Adverse Impact of Bowel Problems on Participants' Lives

#### 3.1.1. The Nature and Burden of Constipation: Psychological Distress

All participants in both the intervention and control groups stated that constipation was the main bowel problem they experienced (other specific bowel problems mentioned were IBS and diverticulitis). The duration of constipation ranged from two months to five years. Three participants recalled that their constipation began around the time of their Parkinson's diagnosis, while two participants perceived an association between their constipation and their Parkinson's medication. Symptoms associated with constipation included flatulence, bloating, nausea, and lethargy and were reported as extremely bothersome. As one participant described:

*Well you don't feel 100% because you're sluggish and I have to strain a lot to get movement. Participant 14, Male, Intervention*

Feeling constipated resulted in participants going to the toilet more frequently and for longer periods, but often without achieving a bowel movement until days later. Stools were often described as being like small pellets which involved straining, pain, and discomfort with some using digital stimulation to encourage a bowel movement.

*The stools are very very hard like round balls … it's very very painful to try and go to the toilet. Participant 6, Male, Intervention*

Participants emphasised that the overall experience of constipation was time consuming and detracted from their ability to perform daily activities. Furthermore, the perception of having “no control” over their bowel movements caused concern for a number of individuals who curtailed social activity specifically due to the burden of constipation. This included going out shopping or taking part in occasions with family and friends. The constant need to be close to a toilet and the corresponding fear of not finding one close by, especially after taking laxatives, required either careful forward planning or deciding simply not to go out at all.

*This is going to sound daft, but you don't go out so often. I'm frightened to go out in case I need to go … you don't know when you're going to need to go to the toilet. That's the big thing. Participant 5, Male, Control*

In summary, the burden of constipation was expressed through a range of emotional, psychological, and social outlets and was continually linked to a perceived negative impact on quality of life. A number of participants felt embarrassed because they had flatulence or had to take laxatives or were generally unhappy about having recurring constipation. One participant described how the discomfort from constipation affected his concentration during meetings at work. Another participant expressed the worry she had about her constipation because this contrasted with previous, very regular bowel movements. While for some participants, coping with the effects of constipation lowered their day-to-day mood, for a participant in the control group it had the effect of affecting him in a deeper way:

*You get depressed. A wee bit of depression set in … even going out for a walk, I've virtually got to make sure that I'm empty before I go out because I don't want to get caught short. Participant 11, Male, Control*

#### 3.1.2. Balancing Solutions with Unpredictable Side-Effects

The psychological burden of constipation was strongly associated with the unpredictable nature of medical aids specific to easing constipation. Most participants in both the intervention and control groups relied on laxatives to contend with their constipation, though some did not use them every day and others reported that they did not always have the desired result of initiating a bowel movement. The unpredictable effect of laxatives was also a concern for many, who described a balancing act between taking laxatives to relieve temporary constipation, against managing the consequences of taking them, for example, experiencing loose stools or diarrhoea. One participant admitted that she was fearful that the physiological influence of laxatives might interfere with the effectiveness of her Parkinson's medications and would use them as a last resort depending on the severity of constipation and stability of Parkinson's combined:

*You're trying to achieve this balance, you're saying, well, on the one hand I'm getting a bit of discomfort in terms of my stomach, my bowels, but in terms of Parkinson's I'm feeling a lot better. So you're trying to do as little as you can to disrupt that. Participant 4, Female, Control *

Implementing dietary changes was mentioned as an alternative approach to dealing with constipation. Examples of dietary changes that participants made included drinking prune juice and eating more fruit, vegetables, and roughage, for example, brown bread and Weetabix. In some instances, participants first implemented dietary changes before deciding to take laxatives or continued to combine the two approaches.

#### 3.1.3. Meeting Educational Needs

The participant group was divided almost equally between those who had received information or advice about bowel problems from specialist healthcare staff and those who had not. In the former group, without exception, participants stated that their bowel problems had been discussed with Parkinson's nurses during clinic appointments. Emerging from participants' accounts was a sense that these discussions were typically quite brief and often initiated by participants themselves. As Participant 13 describes:

*I found that if you bring something up then they [specialist staff] were working things out for you, but I wouldn't say that they told me about them [bowel problems]. Participant 13, Male, Control*

The input from specialist staff often made minimal difference to these individuals who instead preferred to cope with their bowel problems themselves, sometimes with the help of other information sources such as the Internet. Participants also highlighted that discussing bowel problems was not always considered an acceptable thing to do in everyday life and often felt embarrassed at initiating conversations on the subject. For a few participants, this was exacerbated by the lack of someone to share such difficulties with. These individuals reported to value the opportunity to discuss their otherwise “taboo” bowel problems with the study researcher who reduced their anxiety and encouraged them to be honest about their experiences with constipation.

Those who had not received specialist advice reflected that bowel problems were perhaps not a priority for discussion with healthcare staff, including GPs, because the focus was more on how they were coping with the general development of their Parkinson's:

*When you go to see the doctor about [husband's] Parkinson's … they don't have the time. He's more interested in what [husband] can do with his hand and how he's able to stand up, but they never mentioned bowel to me once. Wife of Participant 5, Male, Control*

### 3.2. Positive Experience of Behaviour Adjustments: Experimentation

All participants in the study received lifestyle advice over the 6-week study duration that aimed to help reduce their constipation. Topics included diet, fluid intake, and sitting position, and participants were recommended to monitor their bowel movements with the use of a bowel diary. In general, participants described experiences of increased self-awareness specific to their bowel problems upon implementing the lifestyle advice and often reported direct improvements to the severity of their constipation. This was achieved through a process of experimentation and determination which enabled participants to identify individualised triggers and sensitivities.

#### 3.2.1. Bowel Diaries

All participants stated that they had kept a bowel diary during the study period, with the majority claiming that it was a useful tool to document their bowel habits. Particular reference was made to the fact that participants could easily monitor changes in stool type over time and objectively check if remedial action was needed to improve fluid levels or make dietary changes. This increase in awareness allowed participants to reflect on how their daily behaviours and established routines may impact or relate to their bowel problems. As one participant highlighted:

*… it's not till you start writing things down you realise how many times you go to the toilet, how you do the toilet, what positions you're in. All of a sudden you're challenged to think, well, you've always done it that way, but why? [Participant 13, Male, Control]*

#### 3.2.2. Dietary Advice

Within the study, members of both the intervention and control groups had the opportunity to discuss their current diet with the researcher and, if appropriate, to explore ways in which dietary changes might help to alleviate their constipation. Participants' accounts of their discussions about diet were of two types: those who recounted that they were “already doing the right things” and those who were recommended to make changes. Advice included regularly eating more fruit, vegetables and high fibre foods, adding foods such as yoghurts to lunches, and increasing the frequency of snacks in between meals.

Those participants who did make changes to their diet found them to have a positive impact on their bowel movements and indicated that they had continued with the changes. Participants highlighted the role of partners and family members in encouraging them to incorporate and maintain these dietary modifications.

#### 3.2.3. Fluid Intake

As an integral aspect of exploring dietary behaviour during the study, participants' levels of fluid intake were also explored and encouraged. Some individuals explained that gaining an understanding about the potential relationship between lack of fluid intake and constipation proved enlightening for them. As one participant's wife described:

*I didn't realise it was the liquid that [E] needed to take; it didn't matter so much what I was feeding him up, it was lying in the bowel because there wasn't enough liquid. [Wife of Participant 5, Male, Control]*

A range of approaches were adopted to help maintain higher fluid intake such as filling bottles with water or juice and using these throughout the day, taking water regularly with their medication, or drinking alternatives to water (e.g., soda water, juices, or tea). Two-thirds of participants who had initially increased their fluid intake said that they had maintained this behaviour after participating in the study and reported that this continued to ease their symptoms of constipation. There was a variety of reasons why some participants had not been able to maintain higher fluid levels, including travelling and forgetfulness. However two participants explained that after increasing their fluid intake they had perceived no effect on their constipation and had therefore reverted back to previous levels.

#### 3.2.4. Sitting Position

Those who followed advice about trying to adopt an improved sitting position to facilitate bowel movements found it generally helpful in achieving bowel movements. Specifically, some participants said that it helped reduce straining, prompted mindfulness about sitting up straighter on the toilet, and made sitting on the toilet more comfortable. Over half of the participants reported that they were continuing to use the sitting technique after the 6-week study period [4 intervention; 4 control], and one female participant recounted that her husband had even made her a small wooden stool so that she could elevate her feet when she went to the toilet. Two participants in the control group had not continued to use the technique: one because he did not perceive it to have any effect for him and the other reported difficulty doing so due to mobility aids installed in her bathroom following a recent hip replacement operation.

### 3.3. Abdominal Massage as a Dynamic and Relaxing Tool: Experiential Learning (Intervention Group Only)

Participants in the intervention group were taught abdominal massage and given a DVD at the start of the study and were visited once a week over the 6 weeks to discuss their diet, lifestyle, and how they (or their carer) were getting on with the massage. Generally, as a way of gauging the effect of the massage intervention, participants compared their bowel problems before and after intervention.

Performing abdominal massage produced a variety of effects for participants. Four individuals in the intervention group reported an improvement in their bowel problems during the study period and three saw minimal or no change. For those who reported immediate improvements (sometimes occurring after the very first massage), experiences included reduced or no constipation, more regular bowel movements, less straining, less bloating, and an increased sense of when a bowel movement was going to occur. Changes in stool type and less total time spent on the toilet were also described:

* I don't sit on the toilet for so long. I come out after ten minutes whereas before it was thirty-five minutes. Participant 8, Female, Intervention*

Participants also described that they felt relaxed, comfortable, and generally at ease when receiving abdominal massage. One participant reported: “I felt like falling asleep.” Additionally, a male participant [Participant 3] reflected on how the massage intervention had helped him to deal with his Parkinson's in a wider sense and increase his motivation to engage more fully and positively in managing his symptoms. Further, having the ability to apply the self-massage technique and experience its positive effect reduced the negative impact of constipation and gave him one less thing to deal with on a daily basis:

*It's getting me focused again, I'm a bit more relaxed … in dealing with symptoms as well … I think it's like giving you a tool … [The massage] is a big help. It's something less you're dealing with, you know, because Parkinson's is enough to deal with. Participant 3, Male, Intervention *

Those who reported a continued improvement to their bowel problems were also those who had continued to practice abdominal massage regularly, either by self-massage or given by their husband or wife. Three participants perceived continued improvements to their bowel problems following the “very good” and “very useful” intervention and felt that their bowel habits were “easier” and “more regular” as a result. For two participants this was accompanied by a reduction in laxative use, which resulted in an increase in motivation to reengage with social activities.

Three participants perceived minimal or no changes to their bowel problems even if improvements had been perceived initially. In other words, any improvements that were observed were not maintained, despite continuing to use the massage for the 6-week study duration. Symptoms of constipation remained an issue for these participants, and each continued to take daily laxatives. As Participant 9 describes:

* I'd say at the moment [my constipation] is as bad as it has ever been. Participant 9, Male, Intervention*

Of those who reported little or no improvement to their bowel problems, no one had continued to carry out regular abdominal massage beyond the study period of 6 weeks. However one participant in this group did self-massage if his constipation lasted more than two or three days. Reasons given for ceasing massage were lack of perceived improvement in constipation severity, lack of physical strength in hands and arms (either self or of partner), and changes in daily routine affected by travel.

### 3.4. Abdominal Massage as a Contingency Plan: Hesitation (Control Group Only)

Participants in the control group received advice about their diet and lifestyle once a week over the 6-week study duration. If they wanted, they were also advised and instructed on the abdominal massage by the study researcher* after* the 6-week intervention period was completed at the 10-week assessment session. Thus the control group received less training and no support to implement the abdominal massage. Participants in the control group reported mixed results of performing abdominal massage. One female participant did not practice abdominal massage after being shown, expressing that she felt “guilty” about not doing so [CP12, Female, Control]. Reasons for not continuing were her husband's reticence about applying the technique, using other ways to control constipation and experiencing other health problems. One participant stopping after approximately two weeks due to experiencing pain in his lower abdomen when his wife applied the massage techniques. The experience of abdominal massage was also reported as uncomfortable and awkward for two participants, with minimal perceived impact on constipation. This lack of immediate positive experience or impact of abdominal massage on severity of constipation caused participants to feel hesitant towards using it as a tool to alleviate their bowel problems. One individual further stated that he preferred to focus on the dietary and lifestyle advice he had received (such as increased roughage and varying his position on the toilet), as these changes incurred a reduction in his constipation severity:

*[The massage] was a lot of work, for not a lot of change. I've been concentrating more on the initial comments and remedies [that were] suggested. And they definitely helped, not 100% but maybe 95%. Participant 11, Male, Control*

Of those who gave data, two participants from the control group were still continuing to use the technique at the time of interview. A female participant commented that she continued to use the self-massage technique, despite finding it quite difficult to do and anticipated continued improvement in her technique with practice. Another participant's wife explained that she continued to use massage on her husband if he experienced constipation for three to four days, because she found that this helped to initiate a bowel movement.

## 4. Discussion

The findings of this research confirm previous evidence that PwP can suffer from constipation, which often presents as a frequent and emotionally troublesome nonmotor feature of the disease [2, 7, 32]. The themes from the narratives also align with Kaye et al. (2006) such that many participants felt concerned at the severity of their constipation symptoms and often relied on laxatives to help ease their constipation.

Research has shown that experiencing emotional stress, anxiety, and cognitive impairment may contribute to constipation by overstimulating the sympathetic nervous system, which can result in decreased digestive motility [33, 34]. The majority of participants in this study perceived constipation to have a negative impact on their QoL, experiencing general low mood and fear regarding the consequences of laxative use, which curtailed social activity. Depression in Parkinson's has been well documented in the literature [8, 35] and the present results highlight both the physical and psychological strain of constipation within this population.

Participants described discrepancies and inconsistencies in consultation experiences such that some were alerted to associations between Parkinson's and constipation and others were not. In the latter circumstance, it seemed that emphasis was placed on the motor symptoms of Parkinson's, such as motor complications and motor disability, to the detriment of nonmotor symptoms. Those who had not received direct advice from their Parkinson's nurse (or equivalent) sought out strategies of self-management from alternate sources including the Internet; however the information gathered from these methods is not always as reliable as that from health care professionals. Helping patients realise the likelihood of developing constipation may help them feel more at ease about talking through similar nonmotor symptoms and provides an opportunity to learn simple yet effective strategies such as abdominal massage, which may have significant impact on QoL.

The topic of constipation is often reported as a “taboo” subject within clinical settings, resulting in many PwP feeling embarrassed to talk to health care professionals about their bowel movements [36]. Participants in the present study recalled similar feelings of embarrassment when discussing their constipation with healthcare professionals and thus welcomed the opportunity to openly discuss their bowel movements with the researcher. Indeed, many participants reported feelings of relief at the chance to talk freely about their experiences with constipation. This may in turn reduce stress and anxiety and enable the digestive system to work more effectively [34]. This perspective is likely to reflect both the topical nature of the study-specific conversations and the researcher's encouragement of discussing a potentially sensitive subject.

A number of study-specific tools were also believed to be helpful in relieving the impact of constipation on daily life through means of increasing one's self-awareness of the condition and its exacerbations. For example, using a bowel diary to record bowel movements allowed participants to note the nature of their constipation (e.g., stool frequency, consistency, size, and degree of straining), observe patterns in their preceding nutritional choices, and gain a deeper understanding of their overall Parkinson's health status. Other studies have affirmed diary use as a positive bowel management strategy in the alleviation of constipation and associated symptoms [26]. Increased consumption of liquids was also perceived to reduce severity of constipation. These findings provide support for HCPs to encourage the use of bowel diaries and increased fluid intake as potential constipation aids for PwP, while taking individual preference and lifestyle into consideration.

Abdominal massage was reported as a pleasant and relaxing experience which aligns with previous work [20, 22]. Most participants in the intervention group reported positive impacts both physically (including improved bowel function, reduction in time spent defecating, less straining and bloating, increased completeness of evacuation, and reduced dependence on laxatives) and emotionally, for example, feeling empowered to self-manage their symptoms. This combination of positive visual* and* kinaesthetic feedback may help to explain why the abdominal massage was perceived to be an effective treatment for constipation for these specific participants, as the evidential change in outcomes motivated them to continue.

However some participants did not perceive any improvements in their bowel movements from the abdominal massage over the study period and thus discontinued with abdominal massage sessions. A number of these individuals preferred to rely on the lifestyle advice to ease their constipation and use abdominal massage as a contingency plan if their constipation was particularly bothersome. This reiterates that abdominal massage may not be effective for all individuals who experience constipation and further work is needed to define those in whom it may work or may pose beneficial. Nonetheless, the findings from the present study suggest that abdominal massage may offer an additional treatment option for PwP who have constipation which is noninvasive and few side-effects. This may be an important perception for PwP who do not want to take additional medication to alleviate their constipation.

### 4.1. Limitations

Only a small number of interviews were conducted from the original cohort, which means that the findings presented here must be viewed as one possible description of experiences of individuals who live with Parkinson's and constipation. Furthermore, the participants may represent a biased sample towards those who were not only happy to talk about their treatment but had also perceived benefits from the lifestyle advice and abdominal massage specifically. Further research is therefore required with diverse samples of PwP to extend understanding of the experience of constipation and its treatments in this specific population.

## 5. Conclusion

This study has provided insight into the experiences of PwP who suffer with constipation taking part in a six-week lifestyle advice and abdominal massage programme as an intervention for constipation management. Many participants perceived lifestyle advice and abdominal massage to relieve symptoms and severity of constipation and increase their QoL. Lifestyle advice and abdominal massage (both in combination and separately) may therefore provide effective strategies for constipation management in those who live with Parkinson's and particularly in those who may not wish to rely on multiple medications to alleviate their symptoms of bowel dysfunction. This has implications for clinicians who wish to understand and alleviate the burden of constipation in PwP and also for further research that aims to identify and explore potential interventions for constipation.

This study also highlighted that people who live with Parkinson's and constipation warrant improved education and explanation from HCPs about the nonmotor symptoms of Parkinson's in the goal of holistic and person-centred care. In light of the results presented, HCPs may therefore wish to include lifestyle advice and abdominal massage specifically in their advice to PwP, to potentially relieve the impact and severity of constipation on daily life and increase QoL. However individual tolerability and preference must be considered before any recommendations are made.

## Appendix

### A. Interview Schedule

*Study of the Relief of Constipation Using Abdominal Massage for Patients with Parkinson's Disease*

(1) Can you describe your PD symptoms?
Probes:
  (i) Would you suffer from constipation as a result of PD?
  (ii) Would you suffer from any other bowel problems as a result of PD?
  (iii) Have you ever suffered from faecal incontinence?
(2) What impact has PD had on your life?
(3) What impact has the constipation and/or bowel problems had?
Probes:
  (i) Impact on family life
  (ii) Impact on social life
  (iii) Impact on self-perception
  (iv) Impact on ability to work
  (v) Impact on everyday tasks for example, housework
(4) How do you manage your symptoms of PD?
Probe:
  (i) How do you manage the bowel problems?
  (ii) Have you used laxatives?
(5) What information had you received about PD and bowel problems/constipation?
Probes:
  (i) Information about the causes of bowel problems
  (ii) What symptoms to expect
  (iii) How to alleviate symptoms
  (iv) Sources of information (e.g. GP, PD nurses, Consultant; voluntary groups)
(6) How have you found the massage technique?
Probe:
  (i) How long have you been doing it yourself?
  (ii) What do you think of the technique?
(7) When do you tend to do the massage?
Probes:
  (i) In bed/on toilet/night time
(8) Who does the massage?
Probe:
  (i) Self massage and/or carer and/or partner
  (ii) How do you feel about this?
(9) Do you think a physio or someone trained in massage would be more effective?
Probe:
  (i) Positives and negatives of physio/outside person doing massage
(10) How did you find using the diary?
Probe:
  (i) Was the diary useful? Why?
(11) What did you think of the DVD?
Probe:
  (i) How did you use the DVD?
  (ii) Was the DVD useful? Why?
(12) What did you think of the weekly visits?
(13) Was the programme long enough?
(14) Do you think that the massage helped with your constipation and/or bowel problems?
Probe:
  (i) How did it help?
  (ii) Did it affect your use of laxatives (if applicable?)
  (iii) Has it stabilised your constipation?
  (iv) Has it had any impact on overflow incontinence?
  (v) Has it affected any bladder problems you might have had?
(15) Has the massage helped with any other problems associated with PD?
(16) Can you think of any other way the massage programme could be improved for other PD patients?
Probes:
  (i) Would you use a hand-held massager? Why?
  (ii) Would you like to know of any other exercises that might help your symptoms?
  (iii) Would you attend a group forum or group exercise class showing how to alleviate PD symptoms?
(17) Is there anything else you want to add about the programme?

### B. Themes and Subthemes from Interview Data

(3.1) The adverse impact of bowel problems on participants' quality of life
  (3.1.1) The nature and burden of constipation: psychological distress
  (3.1.2) Balancing solutions with unpredictable side-effects
  (3.1.3) Meeting educational needs
(3.2) Positive experience of behaviour adjustments: experimentation
(3.3) Abdominal massage as a dynamic and relaxing tool: experiential learning (Intervention group only)
(3.4) Abdominal massage as a contingency plan: hesitation (Control Group only)
